# Computational Design and Immunoinformatic Analysis of a Broad-Spectrum Edible Multi-Epitope Vaccine Against Salmonella for Poultry

**DOI:** 10.3390/vetsci13020123

**Published:** 2026-01-28

**Authors:** Lenin J. Ramirez-Cando, Yuliana I. Mora-Ochoa, Jose A. Castillo

**Affiliations:** 1 School of Biological Sciences and Engineering, Yachay University for Experimental Technology and Research (Yachay Tech), Urcuqui 100115, Ecuador; jcastillo@yachaytech.edu.ec; 2 Escuela de Ciencias Agropecuarias y Ambientales, Pontifical Catholic University of Ecuador, Ibarra 100112, Ecuador; yimora@pucesi.edu.ec

**Keywords:** chlorella vulgaris, multi-epitope vaccine, poultry, protein subunit, Salmonella

## Abstract

Salmonellosis remains a persistent threat to global food safety and poultry productivity, compounded by rising antimicrobial resistance. Here, we report the in silico design and immunoinformatic validation of a broad-spectrum, edible multi-epitope vaccine targeting conserved adhesion and biofilm-associated proteins (FimH, AgfA, SefA, SefD, and MrkD) of *Salmonella* spp. Two constructs were engineered by integrating cytotoxic (CTL) and helper (HTL) epitopes with β-defensin-3 (HBD-3) or lipopolysaccharide (LPS) adjuvants, optimized for expression in Chlorella vulgaris. Structural modeling confirmed native-like folding (z-scores −2.58 and −5.22) and high stability indices. Molecular docking and dynamics revealed that the LPS-adjuvanted construct (Construct 2) forms a highly stable complex with Toll-like receptor 3 (HADDOCK score −63.4; desolvation energy −50.2 kcal/mol), indicating potent innate immune activation. Immune simulations predicted strong IgM-to-IgG class switching and durable humoral responses, consistent with effective antigen clearance. Codon optimization achieved high adaptability for algal expression (CAI = 0.93; GC ≈ 65%), supporting scalable microalgae-based production. Compared with current parenteral vaccines, offering a low-cost, non-invasive way to curb Salmonella in poultry, this edible vaccine platform reduces dependence on antibiotics. Our approach, which combines computational vaccinology with a safe-by-design sustainable biomanufacturing perspective, outlines a One Health framework for advancing antimicrobial stewardship and food safety.

## Simple Summary

This research has developed a promising edible vaccine to combat Salmonella infections in poultry, a major source of foodborne illness worldwide. Using advanced computational and immunoinformatic tools, the team designed two multi-epitope vaccine constructs targeting conserved proteins involved in bacterial adhesion and biofilm formation. The vaccines, engineered for expression in the microalga Chlorella vulgaris, incorporate immune-activating adjuvants—β-defensin-3 and lipopolysaccharide (LPS)—to stimulate robust immune responses. Structural modeling and molecular docking revealed that the LPS-based construct (Construct 2) binds strongly to Toll-like receptor 3, suggesting potent innate immune activation. Simulated immune responses showed effective IgM-to-IgG class switching and long-lasting antibody production, indicating strong protection potential. Codon optimization confirmed high expression feasibility in algae, paving the way for scalable, low-cost oral vaccine production. This innovation aligns with One Health principles, aiming to reduce antibiotic use in agriculture, enhance food safety, and mitigate antimicrobial resistance. Experimental trials are underway to validate the vaccine’s efficacy in live poultry.

## 1. Introduction

Salmonellosis, caused by various species of the Salmonella genus, remains one of the most pervasive foodborne infections worldwide, with significant implications for human health, agricultural productivity, and affecting global economies [1]. The genus Salmonella comprises over 2600 serovars, classified into typhoidal (e.g., *S. typhi*, *S. paratyphi*) and non-typhoidal (e.g., *S. typhimurium*, *S. enteritidis*) strains [2,3]. While typhoidal serovars are responsible for enteric fever—a systemic, life-threatening illness endemic to regions with poor sanitation—the non-typhoidal Salmonella (NTS) predominantly causes self-limiting gastroenteritis but poses severe risks to immunocompromised individuals, young children, and the elderly [2,4]. The World Health Organization (WHO, https://www.who.int/data/gho/publications/world-health-statistics, accessed on 21 September 2025) estimates 93.8 million annual cases of gastroenteritis and 11–21 million cases of typhoid fever globally, resulting in approximately 200,000 deaths. Beyond human morbidity, Salmonella infections in livestock, particularly poultry and swine, disrupt food chains and incur billions in annual losses due to contaminated products, trade restrictions, and culling of infected animals [5,6].

The epidemiology of Salmonella infections reflects stark disparities between high-income and low-income regions. Typhoid fever, transmitted via fecal–oral routes, disproportionately affects populations lacking access to clean water and hygiene facilities [6,7]. In contrast, NTS infections in industrialized nations are often linked to contaminated poultry, eggs, and fresh produce, highlighting vulnerabilities in food production systems [2,6]. The rise in invasive non-typhoidal Salmonella (iNTS) diseases in sub-Saharan Africa—where HIV/AIDS, malaria, and malnutrition weaken host immunity—has further complicated the landscape. iNTS strains such as *S. typhimurium* ST313 exhibit high mortality rates (20–25%) due to delayed diagnosis and limited treatment options [7,8,9].

Economically, Salmonella imposes dual burdens: healthcare costs for treating infections and losses from recalls of contaminated foods. In the U.S. alone, annual economic losses exceed $3.7 billion, with poultry-associated outbreaks accounting for 19% of foodborne illnesses [8,10]. Meanwhile, smallholder farmers in developing countries face ruin when livestock infections trigger market bans [6,10,11,12]. These interconnected challenges underscore the need for effective and low-cost preventive strategies that transcend borders and sectors.

The strategies to control salmonellosis have often focused on improving sanitation, food safety protocols, and antibiotic therapies. However, these measures face critical limitations. Sanitation infrastructure remains inadequate in low-resource settings, where typhoid fever thrives, i.e., developing countries in the south [2,5,6,10]. Antibiotic resistance, fueled by the misuse of antimicrobials in agriculture and human medicine, has rendered first-line drugs like ampicillin and ciprofloxacin increasingly ineffective. For instance, multidrug-resistant (MDR) *S. typhi* strains have emerged in South Asia, Latin America, and sub-Saharan Africa, while extensively drug-resistant (XDR) variants resistant to azithromycin and cephalosporins threaten to destabilize outbreak responses [1,11]. In this context, vaccination has emerged as a cost-effective, sustainable tool to reduce disease burden, prevent outbreaks, and mitigate antimicrobial resistance (AMR).

Vaccination offers a proactive solution to salmonellosis by priming the immune system to recognize and neutralize Salmonella before it establishes infection. Unlike antibiotics, vaccines do not contribute to AMR and provide long-term protection, reducing reliance on therapeutic drugs. Current vaccine strategies target both human and animal populations, aligning with the One Health principle that human, animal, and environmental health are interdependent [12,13]. Typhoid vaccines have undergone significant evolution since the early 20th century. The first-generation whole-cell killed vaccines, though effective, caused frequent adverse reactions, leading to their discontinuation. Despite their utility, these vaccines are underutilized in the endemic regions due to cost, cold-chain requirements, and limited protection in children under 2 years. The 2017 prequalification of Typbar-TCV, a Vi-tetanus toxoid conjugate vaccine, marked a breakthrough. Typbar-TCV demonstrates >80% efficacy in infants, induces memory B-cell responses, and is compatible with routine immunization schedules [14,15,16]. In pilot studies across Pakistan, Liberia, and Zimbabwe, Gavi-funded typhoid conjugate vaccine (TCV) rollouts averted 46–63% of typhoid cases [17].

To address the need for broad-spectrum Salmonella control in poultry, this study designed a novel edible multi-epitope vaccine using an integrated in silico approach. Leveraging reverse vaccinology and immunoinformatic tools, the proposed construct is optimized for expression in microalgae, offering a promising One Health-compliant strategy to reduce zoonotic transmission and antimicrobial use.

## 2. Materials and Methods

### 2.1. Data Preparation

We obtained the *Salmonella typhimurium* genome (NCBI accession: L19338) from a study [18]. Within this sequence, the region spanning amino acids from 5622 to 6629 was identified as fimH coding fimbria, which corresponds to the NCBI (https://www.ncbi.nlm.nih.gov/, accessed on 4 April 2025) protein accession AA75420.1. For additional virulence targets, we sourced the following sequences: AgfA (Access code: AAC43599.1), SefA (Access code: QXE98859.1), SefD (Access code: QXE98865.1), PefA (Access code: AOX48419.1), LpfE (Access code: AAA73970.1) and also we use protein MrkD (Access code: AGZ83319.1) of *Klebsiella pneumoniae*, homologous protein for Salmonella fimbriae adhesin. Fimbriae enable Salmonella to adhere to and colonize diverse host cells. The FimH adhesin is a component of the fim operon, which encodes Type I fimbriae. This protein is displayed at the tip of the fimbrial structure and confers upon the bacterium the capacity for cellular adherence, specifically through binding to mannose-containing oligosaccharides. Another significant group is the Sef fimbriae, encoded by the sefABCD operon, which are implicated in virulence and the initial adhesion phase. Certain genes specialized for Salmonella virulence are plasmid-associated; among these is pefA, which encodes fimbriae that mediate adhesion to host cells. The genes for phase 2 flagellar antigens and long polar fimbriae (Lpf) are also plasmid-encoded. Within this system, the lpfE operon contributes to the colonization of the host intestinal epithelium. The gene encoding the Aggressive Factor A (AgfA) facilitates the production of fimbriae involved in intestinal colonization and adhesion, as well as systemic infection within the host. Furthermore, the MrkD protein encodes the adhesin for Gram-negative bacteria and is part of the Mrk operon responsible for the expression of Type 3 fimbriae.

BLASTp analysis was performed for all proteins obtained (https://blast.ncbi.nlm.nih.gov/Blast.cgi?PROGRAM=blastp&PAGE_TYPE=BlastSearch&LINK_LOC=blasthome, accessed on 4 April 2025), using a 100% query coverage criterion and selecting sequences from different *Salmonella* types. We aggregated BLASTp-aligned sequences into a FASTA file for downstream analysis. Subsequently, a multiple sequence alignment was conducted using Clustal Omega version 1.2.4 (https://www.ebi.ac.uk/jdispatcher/msa/clustalo, accessed on 10 April 2025) for each protein group. The alignments were visualized in JalView (https://www.jalview.org/, accessed on 10 April 2025) to determine consensus sequences for each group. Finally, the consensus sequences derived from each group were aligned again using Clustal Omega, and a final consensus sequence was generated and visualized in JalView.

Following the download of the aligned sequence FASTA file, we assessed the protein’s antigenicity using the VaxiJen v2.0 server, applying a threshold score of ≥0.4 to determine suitability for the next stage [19].

Cytotoxic T-lymphocyte (CTL) epitopes were predicted using NetCTL 2.1, an artificial neural network-based server (https://services.healthtech.dtu.dk/services/NetCTL-1.2/, accessed on 25 April 2025). CTL values with a score > 0.75 were selected as valid, following the recommendation of [20]. For the CTLs, the Human leukocyte antigens (HLA) parameters present in the A1, A2, A3, A24, A26, B8, B27, B39, B34, B58, and B62 program were considered, with a length of 9-mer. We evaluated allergenicity using AlgPred2, which integrates BLAST, MERCI, and Maximum Likelihood algorithms for binary classification (allergenic/non-allergenic) based on global prediction scores (https://webs.iiitd.edu.in/raghava/algpred2/, accessed on 24 April 2025). The non-allergenic peptides underwent an antigenicity test using Vaxijen v2.0 with a score ≥ 0.4. Then, a toxicity analysis was conducted through the ToxinPred web server (http://crdd.osdd.net/raghava/toxinpred/, accessed on 25 April 2025) based on support vector machine learning (SVM) to determine whether a protein is toxic or non-toxic [21]. Finally, the resulting CTLs were subjected to an immunogenicity analysis using the IEDB Class I Immunogenicity tool available here: (http://tools.iedb.org/immunogenicity/, accessed on 24 April 2025).

Helper T-lymphocyte (HTL) epitopes were predicted using NetMHCIIpan 4.0 with 15-mer peptide length, consensus method, and 17 major DRB1 alleles (DRB1_0101, 0102, 0103, 0301, 0305, 0401–0405, 0408, 0701, 0801, 0803, 0901, 1001, 1101). Predicted epitopes subsequently underwent sequential validation: allergenicity screening via AlgPred2, antigenicity assessment using VaxiJen v2.0, toxicity evaluation with ToxinPred, and IFN-γ induction analysis through IFNepitope’s combined SVM and Motif approach. Finally, all CTL and HTL epitopes were visually inspected to ensure spatial non-overlap as recommended by [20,22]. Following BLASTp and multiple sequence alignment, consensus sequences were generated for all seven target proteins. Proteins PefA and LpfE were excluded from the final vaccine design as their alignments revealed lower conservation across key Salmonella serovars and/or their consensus sequences yielded antigenicity scores (VaxiJen) below the 0.4 threshold, indicating insufficient immunogenic promise for a broad-spectrum vaccine.

### 2.2. Multi-Epitope Vaccine Construction

We engineered multi-epitope vaccines by linking validated CTL and HTL epitopes (non-allergenic, antigenic, non-toxic) using AAY and GPGPG connectors, respectively. Beta-defensin-3 and LPS adjuvants were fused to the N-terminus via EAAAK spacers. The online server trRosetta (https://yanglab.qd.sdu.edu.cn/trRosetta/, accessed on 5 May 2025) was used for the prediction of the vaccines’ structure using a deep learning approach [23]. ProSA-web server was employed to verify the construct through the z-score, which indicates the overall quality through the deviation of the total energy of the structure (https://prosa.services.came.sbg.ac.at/prosa.php, accessed on 5 May 2025) [24], along with the energy against the number of a.a. map. The Ramachandran plot was obtained through the MolProbity server (http://molprobity.biochem.duke.edu/index.php, accessed on 5 May 2025), which determines the quality and normality of vaccine constructs, Rama-z scores falling in a range between −3 and 3 were considered acceptable [25].

We comprehensively characterized the vaccine constructs through three sequential analytical phases. First, allergenicity profiles were assessed using AlgPred2. Second, antigenicity was evaluated via VaxiJen v2.0, with scores exceeding the 0.4 immunogenicity threshold considered positive. Finally, physicochemical properties were quantified using Expasy’s ProtParam server, analyzing seven key parameters: amino acid composition, molecular weight, theoretical isoelectric point (pI), instability index, aliphatic index, cross-system stability predictions (bacterial/yeast/mammalian), and hydropathy profile via grand average of hydropathy (GRAVY).

### 2.3. Prediction of B Cell Epitopes

For the detection of linear and discontinuous B-cell epitopes against a specific antigen, the ElliPro program (http://tools.iedb.org/ellipro/, accessed on 15 May 2025) was used. ElliPro is based on the three-dimensional structure and provides the resulting score as a Protrusion Index (PI). Recommended specifications from the developer were used to make predictions.

### 2.4. Molecular Docking and Dynamics

For molecular docking studies, we selected TLR3 (PDB: 3ULS) and TLR4 (PDB: 3FXI) as receptor targets. Receptor structures were refined using Swiss-Construct for optimal protein folding. Vaccine ligands comprised two adjuvant-specific designs: Construct 1 (beta-defensin-3 conjugate) and Construct 2 (LPS conjugate). Interaction sites were first mapped via RING v4.0 to identify key residues, followed by protein-protein docking in HADDOCK 2.4. As per HADDOCK protocols, top-ranked clusters (indicating stable complexes) were prioritized based on two criteria: (1) HADDOCK scores (more negative values denoting superior binding), and (2) Z-scores (−3 to 3 range) identifying statistically significant interactions [26]. The structures obtained through docking are visualized using the LigPlot+ v2.2.8 software.

Molecular dynamic simulation (MDS) and normal mode analysis (NMA) of the docked complexes were conducted with RStudio V 4.5 and bio3d version 2.4.1.9000 package to assess conformational stability, deformability, and atomic fluctuations.

### 2.5. Immunological Simulation of Vaccine Design

We used the C-ImmSim server to simulate the immune response to both constructs. The tool models a virtual vaccination protocol, predicting the immune response to epitopes within the given sequences (https://kraken.iac.rm.cnr.it/C-IMMSIM/index.php?page=1, accessed on 15 May 2025) [22]. The simulation steps were modified to three constructs to 300 steps.

### 2.6. Codon Optimization and In Silico Cloning

To enhance gene expression within the selected biological host, the nucleotide sequences for the multi-epitope vaccine constructs underwent codon optimization. This process was performed using the VectorBuilder platform (https://en.vectorbuilder.com/tool/codon-optimization.html accessed on 18 May 2025), which tailors the gene’s codon usage to match the preferred translational machinery of the target organism—in this case, the green alga Chlamydomonas reinhardtii. Key parameters for successful expression, including the Codon Adaptation Index (CAI) and GC content, were calculated. A high CAI (theoretically approaching 1.0) indicates optimal alignment with the host’s tRNA abundance, promoting translational efficiency, while an appropriate GC content ensures stable mRNA secondary structure and supports transcriptional fidelity within the algal system.

For the subsequent in silico cloning, two established plant binary vectors, pGreen 0029 and pGreenII 0179, were selected from the SnapGene database. These vectors are well-suited for plant and algal transformation due to their T-DNA border regions and modular design. The optimized gene constructs were then virtually inserted into these plasmid backbones using Benchling (https://www.benchling.com/, accessed on 18 May 2025), a cloud-based informatics platform for molecular biology. The cloning strategy was designed to utilize the EcoRI and BamHI restriction sites, creating compatible sticky ends for precise directional insertion into the vector’s multiple cloning site. This simulated cloning prepares the constructs for eventual transformation and expression in the model microalga Chlorella vulgaris, establishing a workflow for recombinant protein production in an edible plant-based system.

## 3. Results

### 3.1. Construct Design

After a comprehensive pre-analysis we selected a cohort of 5 proteins (FimH, SefA, SefD, AgfA, and MrkD proteins), a comparative sequence analysis was conducted on these 5 proteins from different Salmonella serovars. BLASTp alignment analysis enabled the identification of conserved regions within each protein. From these alignments, consensus sequences were generated, representing the most frequent amino acid sequences at the analyzed positions. These consensus sequences served as the foundation for designing the vaccine peptides, with the goal of ensuring broad coverage against diverse Salmonella strains is presented in FASTA format:

(>Consensus_FimH_AgfA_SefA_SefD_MrkD M++NQY++++++FIV+++MLI+DFW+FCNMRKSASAVA+LAL++CRVVVPPDLPVGSVILTRDWTMSAPGGASYRCGSAHAAGFVAKIVS++++++GNK++S++++G+G++F+R+G++++I+++DVX+++++NT++Y+++G+++++++IKTAEVQAAVTIAAQNTTSA+WSQD++F+GPAVA+GQKVGTLSITATGPHNS+L+++NMN+D+++S+++VSI+GAGKGASV+GGVA+VDLPFVDG++QP+FRNISTSFSGRLATSTTAIQGANINDQANTGI+GLAGWRVASDGSPLQFN++YTVGRLNNQETRYSQ+TLNVPV++FGKSTLP++++T++++S+M++AGTFTATFY+QQYQN)

For the prediction of CTL epitopes, the NetCTL 1.2 tool was used, yielding a total of 3 peptides. Table 1 summarizes the characteristics of the predicted CTL peptides, including their position within the consensus sequence, the HLA supertype they bind to, the peptide sequence, length, prediction method, NetCTL 1.2 score, and predictions for antigenicity, allergenicity, toxicity, and immunogenicity. The NetCTL 1.2 scores reflect the likelihood of the peptide being processed and presented by class I MHC.

An HTL epitope was predicted and Table 1 displays the characteristics of the predicted HTL peptides, including their position, the class II MHC allele they bind to, the peptide sequence, length, NetMHCIIpan 4.0 results (which include the %Rank-EL, representing the peptide binding percentile, and the Bind Level, indicating the peptide’s binding affinity to the allele), as well as predictions for antigenicity, allergenicity, toxicity, and IFN-γ production.

**Figure 1:**
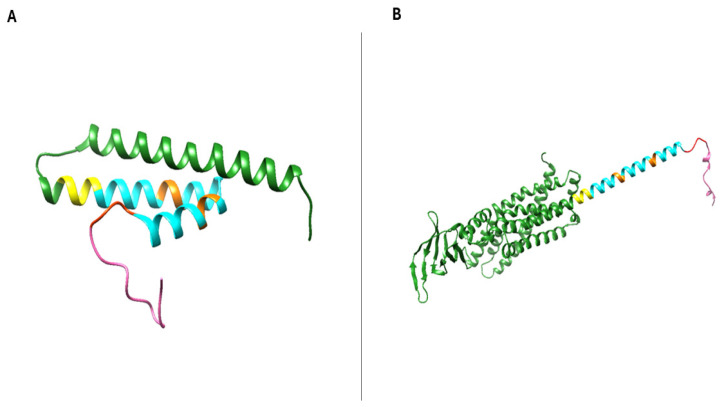
(**A**) Construct One prediction. Color code: Beta-defensine (colour green), EAAAK (colour yellow), CTLs (colour cian), AAY (colour orange), HTL (colour pink) y GPGPG (colour red); and (**B**) Construct Two prediction. Color code:LPS (colour green), EAAAK (colour yellow), CTLs (colour cian), AAY (colour orange), HTL (colour pink) y GPGPG (colour red).

The construction of two multi-epitope vaccine constructs combined the predicted CTL and HTL peptides with adjuvants to enhance the immune response (Table 2). The sequence of Vaccine Construct One, incorporating the beta-defensin adjuvant, is as follows (Figure 1A).

CRFPHIAIGKCATFISCCGRAYEVDALNSVRTSPWLLAPGNNPHEEAAAKNQYFIVMLIAAYIVMLIDFWFAAYYFIVMLIDFGPGPGTRDWTMSAPGGASYR

Meanwhile, Construct Two (Figure 1B), which includes an LPS (lipopolysaccharide) adjuvant, displays the following sequence:

MQPFGVLDRYIGKTIFTTIMMTLFMLVSLSGIIKFVDQLKKAGQGNYDALGAGMYTLLSVPKDIQIFFPMAALLGALLGLGMLAQRSELVVMQASGFTRMQVALSVMKTAIPLVLLTMAIGEWVAPQGEQMARNYRAQAMYGGSLLSTQQGLWAKDGNNFVYIERVKGDEELAGISIYAFNDQRRLQSVRYAASAKFDPEHKVWRLSQVDESDLQNPKQITGSQTVSGTWKTNLTPDKLGVVALDPDALSISGLHNYVKYLKSSGQDAGRYQLNMWSKIFQPLSVAVMMLMALSFIFGPLRSVPMGVRVVTGISFGFVFYVLDQIFGPLTLVYGIPPIVGALLPSASFFLISLWLMLRKSEAAAKNQYFIVMLIAAYIVMLIDFWFAAYYFIVMLIDFGPGPGTRDWTMSAPGGASYR

For each construct, linear and discontinuous B-cell epitopes were predicted to identify regions of the vaccine that may be recognized by antibodies. Table 2 presents the predicted B-cell epitopes for Construct One, including their ID number, protein chain, start and end positions, peptide sequence, number of residues, and prediction score. Similarly, Table 3 presents the corresponding B-cell epitope predictions for Construct Two. The discontinuous B-cell epitopes specify the amino acid residues comprising each epitope, the total number of residues, and the prediction score. Discontinuous epitopes are formed by amino acid residues that are not consecutive in the protein’s primary sequence but are spatially proximate in its three-dimensional structure.

Subsequently, the physicochemical properties of the vaccine constructs were analyzed to characterize their behavior and stability. Table 4 presents these properties for both Construct One and Construct Two, and the data indicate that both constructs are stable and suitable for the next step.

Table 5 presents the antigenicity and allergenicity analysis for both constructs, assessing their immunogenic potential and safety. Based on the AlgPred2 threshold (ML Score ≥ 0.5 for allergen prediction), the two constructs exhibited distinct profiles. Construct One demonstrated high antigenicity (score: 0.9095), indicating a strong potential to elicit adaptive immunity; however, its borderline allergenicity score (0.43) warrants caution. In contrast, Construct Two showed moderate antigenicity (score: 0.4785) but a negligible allergenicity score (0.09), positioning it as a safer candidate with an acceptable immunogenicity profile.

### 3.2. Constructs’ Assessment and Structural Validation

**Figure 2:**
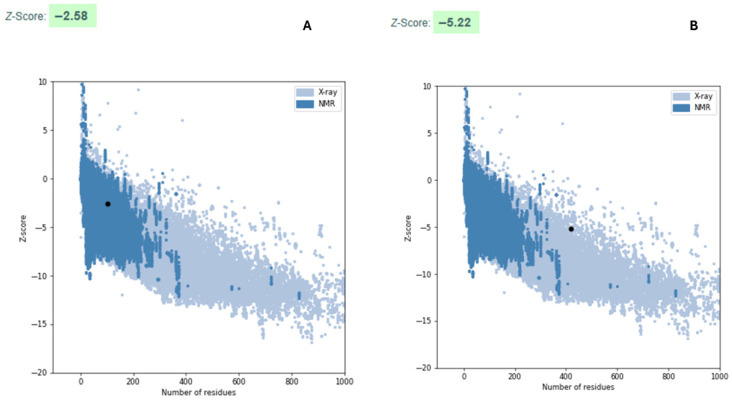
Quality of predicted constructs. (**A**) Construct One and (**B**) Construct Two.

The quality of both constructs was evaluated to determine their naturalness or proximity to a natural/native protein. Construct One exhibited a z-score of −2.58, and Construct Two a z-score of −5.22 (see Figure 2), both within the range characteristic of native proteins. The energy plots for Construct One and Construct Two, corresponding to the *i*-th position of the amino acid sequences, reflect their structural similarity to a native protein, as Figure 2 also indicates.

**Figure 3:**
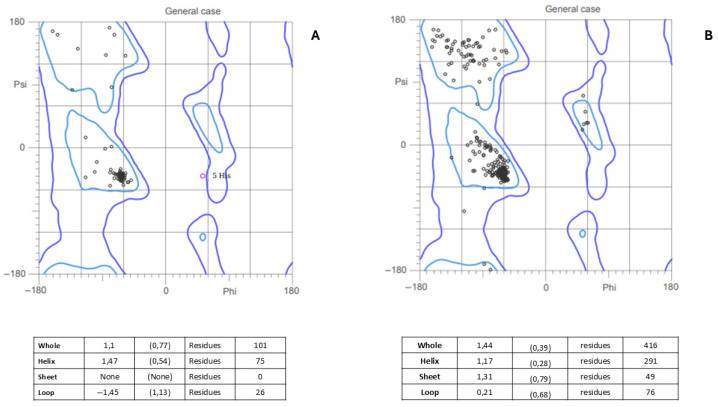
Ramachandran plot and Z-score analysis, interpretation: bad |Rama-Z| > 3; suspicious 2 < |Rama-Z| < 3; good |Rama-Z| < 2. (**A**) Construct One and (**B**) Construct Two.

To assess the structural quality of the vaccine constructs, a Ramachandran plot analysis was performed. This analysis evaluated the distribution of the phi (φ) and psi (ψ) dihedral angles of amino acid residues in the three-dimensional protein structure. The Rama-Z score was used to quantify construct quality. Both constructs exhibited Rama-Z scores between −3 and 3, within the accepted quality metric. Graphically, Construct One displayed very few outliers, while Construct Two showed a higher proportion of residues within allowed regions and minimal outliers, as shown in Figure 3. Both constructs demonstrate high-quality structures, as most residues are concentrated in favored regions.

The docking results, detailed in Table 6, reveal distinct interaction profiles between the vaccine constructs and Toll-like receptors. Specifically, Construct Two demonstrated superior binding affinity and stability with TLR3, evidenced by its significantly lower HADDOCK score (−63.4 vs. −31.5 for Construct One) and favorable energy contributions—such as an electrostatic energy of −231.9 kcal/mol—which suggests robust charge complementarity at the binding interface. In contrast, Construct One exhibits non-physiological binding to TLR4 (positive HADDOCK score: 88.2) due to catastrophic restraint violations (2998.3) and anomalously high electrostatic energy (−578.3), likely reflecting computational artifacts. While Construct Two shows moderate TLR4 affinity (−37.8), its TLR3 interaction is prioritized as the most viable, combining low RMSD variability, strong hydrophobic packing (desolvation energy: −50.2), and minimal violations.

**Figure 4:**
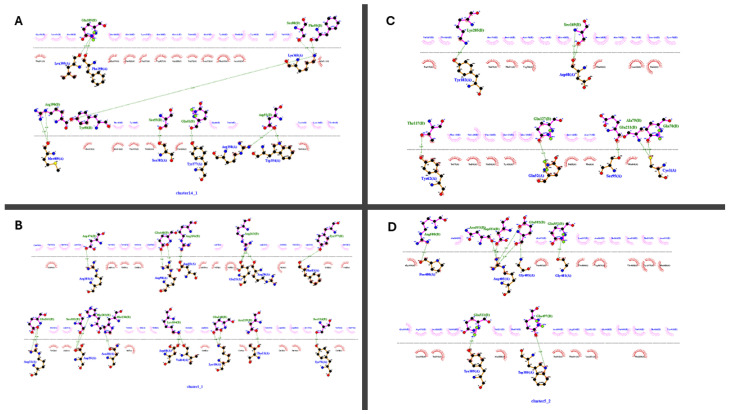
Dimplot of protein complex of TLR receptor domains and vaccine-adjuvant constructs. (**A**) TLR 3–Construct One, (**B**) TLR 4–Construct One, (**C**) TLR 3–Construct Two, and (**D**) TLR 4–Construct Two.

Construct One’s TLR4 results warrant re-evaluation for constructing errors, whereas Construct Two emerges as the lead candidate for TLR3-mediated immune activation, pending experimental validation of binding stability and biological efficacy (see Figure 4).

**Figure 5:**
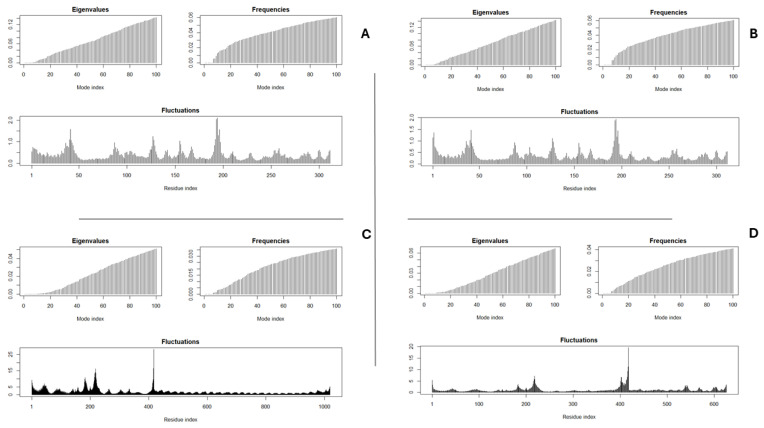
NMA results for (**A**) Molecular Dynamics of Construct One and TLR 4 receptor cluster, (**B**) Molecular Dynamics of Construct Two and TLR 3 receptor cluster, (**C**) Molecular Dynamics of Construct One and TLR 4 receptor cluster and (**D**) Molecular Dynamics of Construct Two and TLR 3 receptor cluster.

NMA conducted through RStudio evaluated deformability, flexibility, and atomic interactions of docked complexes. Deformability plots indicated localized flexibility at hinge 240 points, while eigenvalues suggested stable conformations (see Figure 5). Covariance matrices and elastic network constructs revealed coordinated and stiff atomic interactions, supporting the structural robustness of vaccine-receptor complexes. Eigenvalues and fluctuations in Figure 5 show that there is a soft stiffness in the complex, favorable to the expected dynamic effect.

### 3.3. Immunological Validation

**Figure 6:**
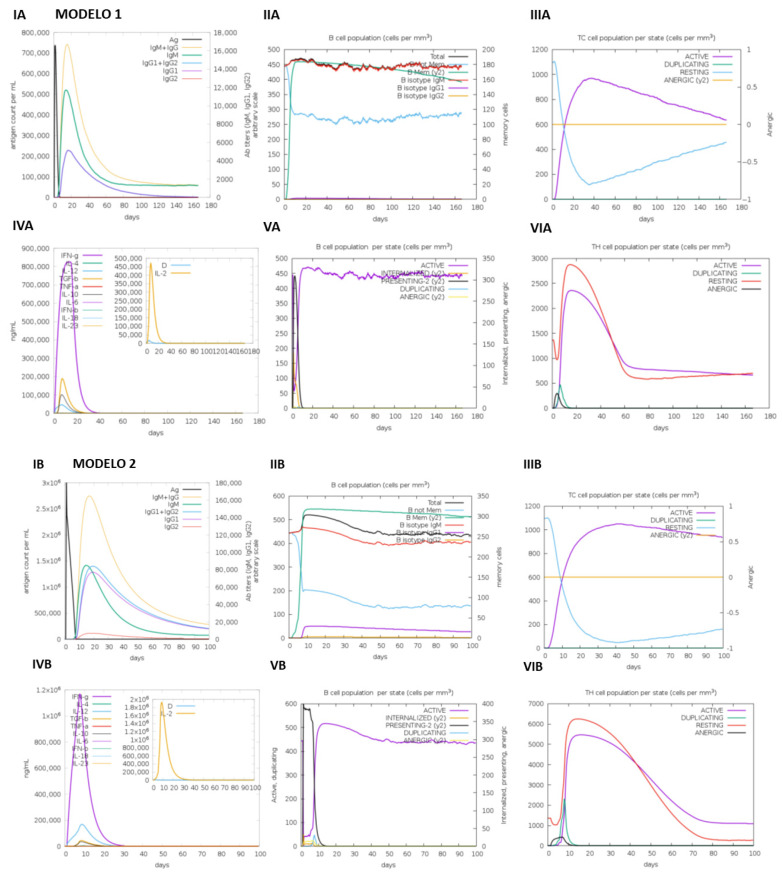
In silico simulated immune response profiles for the multi-epitope vaccine constructs. (**A**) Construct One (β-defensin-adjuvanted). The simulation was performed with a volume of 20 over 500 steps, administering 1000 antigen units alongside 100 units of β-defensin adjuvant. The panel illustrates the characteristic immune activation: an initial peak of IgM followed by sustained production of IgG subclasses (IgG1 and IgG2), concomitant with a decline in antigen levels, indicating effective immune clearance. (**B**) Construct Two (LPS-adjuvanted). The simulation was conducted with a volume of 30 over 300 steps, using a higher dose of 3000 antigen units with 300 units of LPS adjuvant. This regimen induced a more robust and prolonged humoral response. The panel shows a pronounced IgM-to-IgG switch, with enhanced peak magnitude and persistence of IgG isotypes, suggesting stronger and potentially more durable immunity compared to Construct One. For both panels, the left graph displays antibody titers (IgM, IgG1, IgG2), the center graph tracks antigen concentration, and the right graph shows levels of key cytokines (IFN-γ, IL-2, IL-10).

Finally, an immunological simulation was conducted to profile the adaptive immune response elicited by the two vaccine constructs, with the results summarized in Figure 6. For Construct One, simulation parameters were set to a volume of 20, 500 steps, and an injection of 1000 antigens with 100 units of its beta-defensin adjuvant. The simulation revealed the characteristic immune activation: an initial peak of IgM was followed by a clear class switch to sustain IgG (IgG1 and IgG2) production, coupled with a concurrent decline in antigen levels, indicating effective immune clearance (Figure 6A).

In contrast, Construct Two was simulated under different conditions—volume of 30, 300 steps, and a higher dose of 3000 antigens with 300 units of its LPS adjuvant. This regimen induced a more robust and prolonged humoral response. As shown in Figure 6B, the IgM-to-IgG switch remained evident; still, both the peak magnitude and the persistence of IgG isotypes were enhanced, suggesting stronger and potentially more durable immunity. The distinct kinetic profiles in Figure 6B underscore that Construct Two, adjuvanted with LPS, may provoke a superior adaptive immune response.

### 3.4. Cloning Optimization

**Figure 7:**
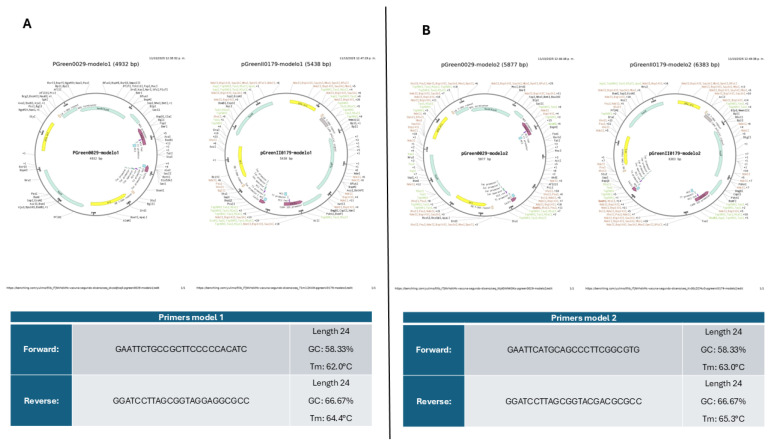
In-silico cloning. (**A**) Cloning into pGreen 0029 and pGreenII 0179 vectors with Construct One, and (**B**) Cloning into pGreen 0029 and pGreenII 0179 vectors with Construct Two.

The optimization of the multi-epitope vaccine designs for the construct organism yielded a Codon Adaptation Index (CAI) of 0.90 for Construct One and 0.93 for Construct Two. Both results exceed 0.9, indicating that the designs of both constructs have a high potential for expression in the host organism. Furthermore, the guanine (G) and cytosine (C) content for each construct was 65.06% for Construct One and 64.84% for Construct Two (see Figure 7). For the primer design, the restriction sites for the enzymes EcoRI and BamHI were added to each construct. These sites were identified within the pGreen 0029 and pGreenII 0179 plasmids and were utilized for the in silico cloning procedure.

## 4. Discussion

Salmonellosis remains a formidable challenge in poultry production, particularly in broilers, where non-typhoidal Salmonella (NTS) serovars cause substantial economic losses and pose zoonotic risks [1,4]. The emergence of multidrug-resistant strains underscores the urgent need for innovative vaccines that circumvent antibiotic reliance [11]. This study presents a computationally designed multi-epitope vaccine targeting conserved adhesion and biofilm-associated proteins (FimH, SefA, SefD, AgfA, MrkD) critical for Salmonella pathogenesis. By leveraging bioinformatics tools, we address key gaps in traditional vaccine strategies, such as narrow serovar coverage and safety concerns, while aligning with One Health principles that bridge animal and human health [1,10,12].

The selection of epitopes from conserved regions of virulent proteins is a strategic strength [27,28]. FimH and AgfA, for instance, mediate host cell adhesion and biofilm formation, making them prime targets for disrupting colonization [16,18,29]. The integration of CTL and HTL epitopes connected via AAY/GPGPG linkers ensures simultaneous activation of cellular and humoral immunity, essential for combating intracellular pathogens like Salmonella [20]. The use of beta-defensin (Construct One) and LPS (Construct Two) as adjuvants reflects a nuanced approach: beta-defensin enhances mucosal immunity, while LPS, a TLR4 agonist, potentiates innate responses. However, LPS’s inherent pyrogenicity necessitates careful safety evaluations; we partially address allergenicity predictions (Construct Two: ML score 0.09, non-allergenic).

Both vaccine constructs demonstrated structural robustness, with z-scores and Ramachandran plots indicative of native-like folding. Construct Two’s superior TLR3 binding (HADDOCK score: −63.4 ± 4.9) highlights LPS’s role in stabilizing interactions with innate immune receptors, though its TLR4 affinity (−37.8 ± 13.7) was unexpectedly moderate. Conversely, Construct One’s TLR4 docking yielded non-physiological scores, likely due to steric clashes or suboptimal adjuvant-receptor pairing. These findings emphasize the importance of adjuvant selection in modulating receptor specificity. Furthermore, NMA performed using the bio3D package confirmed that the Multi Epitope Vaccine construct possesses structural stability and limited flexibility, characteristics favorable for preserving epitope integrity during antigen processing. Furthermore, protein–protein ligand docking simulations demonstrated favorable binding affinities between the MEV and key avian immune receptors, including TLR3 and TLR4 (Table 6 and Figure 5). These interactions support the construct’s potential to engage both innate and adaptive immune pathways, thereby eliciting broad and effective immune responses.

The C-ImmSim simulations revealed distinct immune dynamics. Construct Two, with higher antigen doses (3000 vs. 1000) and LPS, induced prolonged IgG1/IgG2 responses, suggesting durable humoral immunity. The IgM-to-IgG class switch observed in both constructs aligns with adaptive immune maturation, critical for long-term protection [17,22]. For broilers, where rapid seroconversion is vital, Construct Two’s robust antigenicity and low allergenicity make it a promising candidate. However, Construct One’s borderline allergenicity warrants caution, though its beta-defensin component could enhance mucosal immunity in gut-associated lymphoid tissues, a key battleground for Salmonella.

The successful in silico cloning and optimization of both vaccine constructs underscore their potential for practical application and scalable production. Codon optimization for the microalgal host Chlorella vulgaris yielded exceptionally high Codon Adaptation Index (CAI) values of 0.90 and 0.93 for Constructs 1 and 2, respectively, indicating a high likelihood of efficient protein expression. Furthermore, the balanced GC content of approximately 65% for both constructs supports transcript stability and translational efficiency. As validated in Figure 7, the gene sequences were successfully inserted into the pGreen 0029 and pGreenII 0179 plant binary vectors using EcoRI and BamHI restriction sites. The design of specific primers with high melting temperatures and GC content ensures a robust strategy for future molecular validation compared with previous research [20,30]. This comprehensive computational workflow confirms the feasibility of producing these edible vaccine candidates in a microalgae-based system, paving the way for a cost-effective and scalable oral delivery platform in poultry.

While this in silico framework provides a cost-effective and hypothesis-driven foundation, experimental validation remains essential. Controlled in vivo studies in poultry are required to confirm the vaccine’s immunogenicity, safety, and cross-serovar protection. Our laboratory has initiated the experimental phase, encompassing vaccine production and comprehensive in vivo immunological evaluation to substantiate these computational predictions. The use of LPS, though effective, may require detoxification (e.g., monophosphorylate lipid A derivatives) to mitigate inflammatory risks. Additionally, the vaccine’s expression in microalgae—a proposed feature of research—must be optimized for scalability and oral delivery in poultry through an innovative drug delivery system. A successful vaccine could reduce Salmonella colonization in broilers, minimizing vertical transmission and contamination of poultry products. This aligns with global food safety initiatives and antimicrobial resistance (AMR) mitigation efforts and in line with Zero Hunger (SDG 2), Good health and well-being (SDG 3). Economically, curtailing outbreaks would alleviate losses from culling, recalls, and trade restrictions, benefiting smallholder farmers and industrial producers alike.

While this computational study provides a strong, hypothesis-driven foundation, it is not without limitations, which define clear paths for future research.

The promising in silico predictions necessitate rigorous in vivo validation. Our initiated laboratory work focuses on producing the vaccine candidates and evaluating their immunogenicity, safety, and protective efficacy against multiple serovars in poultry challenge models. The immunogenic strength of native LPS is counterbalanced by its reactogenicity. Future iterations should explore detoxified analogs, such as monophosphoryl lipid A, to maintain adjuvanticity while minimizing inflammatory risk.

The successful expression of the recombinant antigen in Chlorella vulgaris must be empirically confirmed and optimized for yield. Furthermore, integrating the antigen into an effective oral delivery system—such as algal biomass—is critical for practical field application. Immune simulations, though insightful, are approximations. The actual immune response in broilers may vary due to genetic factors, microbiota, and management conditions not captured in silico.

## 5. Conclusions

This study found that Construct 2, which uses a detoxified LPS adjuvant, is the superior candidate. It showed a high binding affinity for the poultry-relevant TLR3 receptor (HADDOCK score: −63.4), whereas Construct 1 (with a beta-defensin adjuvant) showed non-physiological binding to TLR4. Construct 2 also combines a low allergenic potential (score: 0.09) with a 51% greater B-cell epitope coverage than Construct 1, indicating it is both safer and more immunogenic. Immune simulations further supported its promise, predicting a strong, prolonged IgG response and efficient antigen clearance. Together, these results position Construct 2 as a broad-spectrum vaccine candidate capable of overcoming the limited serovar coverage of current poultry vaccines.

The promising feasibility of microalgae-based oral vaccine production establishes a strong foundation for future development. The path forward now focuses on the essential biological studies needed to bring this innovation to fruition. Ultimately, successfully translating this design holds the potential to revolutionize poultry health management while making significant strides toward key One Health objectives: curbing antibiotic use, ensuring food security, and mitigating the public health threat of antimicrobial resistance.

## Figures and Tables

**Table 1:** CTL and HTL, prediction and validation analysis.

CTL														
Position	Super Type	Peptide	Length	Method	NetCTL 1.2	Antigen Prediction			Allergen Prediction		Toxicity Prediction		Immunity	
2	A2	NQYFIVMLI	9	COMB	0.8353 <−E	0.6194	Antigen		0.12	Non-Allergen	−0.98	Non-Toxin	0.13038	
6	A24	IVMLIDFWF	9	COMB	1.4859 <−E	3.0250	Antigen		0.2	Non-Allergen	−1.24	Non-Toxin	0.31054	
4	A24	YFIVMLIDF	9	COMB	1.4653 <−E	1.7896	Antigen		0.17	Non-Allergen	−1.09	Non-Toxin	0.02858	
**HTL**														
**Position**	**Allele**	**Peptide**	**Length**	**NetMHCIIpan 4.0–Identity-** **%Rank-EL**		**Bind Level**	**Antigen** **Prediction**		**Allergen** **Prediction**		**Toxicity** **Prediction**		**IFN-γ Prediction**	
45	DRB1_0901	TRDWTMSAPGGASYR	15	Consensus 1–360	0.9	≤SB *	10.900	Antigen	0.29	Non-Allergen	−0.64	Non-Toxin	+	0.12465985

* SB = Strong Binding.

**Table 2:** Linear and discontinuous epitopes predictions for Construct One.

Predicted Linear Epitope(s)						
No	Chain	Start	End	Peptide	Number of Residues	Score
1	A	1	14	CRFPHIAIGKCATF	14	0.782
2	A	86	103	GPGTRDWTMSAPGGASYR	18	0.737
3	A	32	46	TSPWLLAPGNNPHEE	15	0.721
**Predicted Discontinuous Epitope(s)**						
**No**	**Residues**				**Number of Residues**	**Score**
1	A:D91, A:T93, A:M94, A:S95, A:A96, A:P97, A:G98, A:G99, A:A100, A:S101, A:Y102, A:R103				12	0.843
2	A:C1, A:F3, A:P4, A:H5, A:I6, A:A7, A:I8, A:G9, A:K10, A:C11, A:A12, A:T13				12	0.782
3	A:T32, A:S33, A:P34, A:W35, A:L36, A:L37, A:A38, A:P39, A:G40, A:N41, A:N42, A:P43, A:H44, A:E45, A:E46				15	0.721
4	A:G86, A:P87, A:G88, A:T89, A:R90				5	0.577

**Table 3:** Linear and discontinuous epitopes predictions for Construct Two.

Predicted Linear Epitope(s)						
No	Chain	Start	End	Peptide	Number of Residues	Score
1	A	368	418	YFIVMLIAAYIVMLIDFWFAAYYFIVMLIDFGPGPGTRDWTMSAPGGASYR	51	0.876
2	A	28	58	SLSGIIKFVDQLKKAGQGNYDALGAGMYTLL	31	0.781
3	A	1	10	MQPFGVLDRY	10	0.74
4	A	176	234	SIYAFNDQRRLQSVRYAASAKFDPEHKVWRLSQVDESDLQNPKQITGSQTVSGTWKTNL	59	0.732
5	A	135	145	YRAQAMYGGSL	11	0.677
6	A	329	336	LTLVYGIP	8	0.644
7	A	262	268	KSSGQDA	7	0.556
8	A	95	98	SGFT	4	0.508
**Predicted Discontinuous Epitope(s)**						
**No**				**Residues**	**Number of Residues**	**Score**
1				A:S410, A:A411, A:P412, A:G413, A:G414, A:A415, A:S416, A:Y417, A:R418	9	0.986
2				A:D406, A:W407, A:T408, A:M409	4	0.968
3				A:N216, A:P217, A:K218, A:Q219	4	0.94
4				A:W385, A:F386, A:A387, A:A388, A:Y389, A:Y390, A:F391, A:I392, A:V393, A:M394, A:L395, A:I396, A:D397, A:F398, A:G399, A:P400, A:G401, A:P402, A:G403, A:T404, A:R405	21	0.925
5				A:M1, A:Q2, A:P3, A:F4, A:G5, A:V6, A:L7, A:D8, A:R9	9	0.754
6				A:Y368, A:F369, A:I370, A:V371, A:M372, A:L373, A:I374, A:A375, A:A376, A:Y377, A:I378, A:V379, A:M380, A:L381, A:I382, A:D383, A:F384	17	0.735
7				A:M25, A:S28, A:L29, A:S30, A:G31, A:I32, A:I33, A:K34, A:F35, A:V36, A:D37, A:Q38, A:L39, A:K40, A:K41, A:A42, A:G43, A:Q44, A:G45, A:N46, A:Y47, A:D48, A:A49, A:L50, A:G51, A:A52, A:G53, A:M54, A:Y55, A:T56, A:L57, A:L58, A:M131, A:A132, A:Y135, A:R136, A:A137, A:Q138, A:A139, A:M140, A:Y141, A:G142, A:G143, A:S144, A:L145, A:K155, A:D156, A:G157, A:N158, A:N159, A:F160, A:R165, A:K167, A:G168, A:D169, A:E170, A:E171, A:S176, A:I177, A:Y178, A:A179, A:F180, A:N181, A:D182, A:Q183, A:R185, A:L186, A:Q187, A:S188, A:V189, A:R190, A:Y191, A:A192, A:A193, A:S194, A:A195, A:K196, A:F197, A:D198, A:P199, A:E200, A:H201, A:K202, A:V203, A:W204, A:R205, A:L206, A:S207, A:Q208, A:V209, A:D210, A:E211, A:S212, A:D213, A:L214, A:Q215, A:I220, A:T221, A:G222, A:S223, A:Q224, A:T225, A:V226, A:S227, A:G228, A:T229, A:W230, A:K231, A:T232, A:N233, A:L234, A:K262, A:S263, A:S264, A:G265, A:Q266	116	0.707
8				A:L329, A:T330, A:V332, A:Y333, A:G334, A:I335, A:P336, A:V339	8	0.652
9				A:S95, A:G96, A:F97, A:T98, A:Q101	5	0.508

**Table 4:** Predicted physicochemical properties of Constructs.

Predicted Properties	One	Two
Number of amino acids	103	418
Molecular weight	11,431.29	46,187.3
Theoretical pI	6.89	9.3
Instability index	24.88	24.26
Aliphatic index	84.47	101.96
Grand average of hydropathicity (GRAVY)	0.352	0.356

**Table 5:** Antigenicity and allergenicity predictions.

Construct One				Construct Two			
Antigenicity		Allergenicity		Antigenicity		Allergenicity	
Score	0.9095	ML Score	0.43	Score	0.4785	ML Score	0.09
Prediction	Antigen	Prediction	Non-Allergen	Prediction	Antigen	Prediction	Non-Allergen

**Table 6:** Molecular docking analysis of constructs against TLR and TLR 4 receptors.

Parameters	TLR 3		TLR 4	
	Construct One	Construct Two	Construct One	Construct Two
HADDOCK score	−31.5 ± 4.3	−63.4 ± 4.9	88.2 ± 15.7	−37.8 ± 13.7
Cluster size	19	4	49	5
RMSD from the overall lowest-energy structure	2.6 ± 0.9	11.1 ± 0.1	1.5 ± 0.1	3.2 ± 2.9
Van der Waals energy	−73.8 ± 4.4	−88.6 ± 7.5	−78.1 ± 3.4	−73.1 ± 13.3
Electrostatic energy	−110.8 ± 10.7	−231.9 ± 10.7	−578.3 ± 23.1	−204.6 ± 40.3
Desolvation energy	−31.9 ± 2.2	−50.2 ± 2.4	−17.9 ± 3.1	−50.8 ± 6.8
Restraint violation energy	963.7 ± 79.9	1218.0 ± 59.9	2998.3 ± 110.1	1269.9 ± 103.0
Buried Surface Area	2463.3 ± 135.1	3059.4 ± 111.8	3791.3 ± 109.6	2548.9 ± 206.6
Z-Score	−2.1	−2.1	−1.6	−1.6

## Data Availability

The original contributions presented in this study are included in the article. Further inquiries can be directed to the corresponding author.
